# A Comparison of the Effects of Epidural Levobupivacaine and Morphine for Postoperative Analgesia Following Major Abdominal Surgery: A Randomized Controlled Trial

**DOI:** 10.7759/cureus.34900

**Published:** 2023-02-12

**Authors:** Aravindan Rangapriya, Rajagopalan Venkatraman, Mani Karthik, Anandpandi Preethi

**Affiliations:** 1 Anaesthesiology, Sri Ramaswamy Memorial (SRM) Institute of Science and Technology, Chennai, IND

**Keywords:** visual analog scale, postoperative analgesia, morphine, levobupivacaine, epidural analgesia

## Abstract

Objectives

Epidural analgesia remains the cornerstone of pain management following laparotomy. Local anesthetics used in epidural analgesia provide good analgesia but may result in hypotension and/or motor blockade. Morphine, a long-acting opioid, can also be used epidurally to provide analgesia. Morphine used epidurally will cause fewer hemodynamic disturbances and no motor blockade. Hence, we compared the efficacy, hemodynamic parameters, and motor blockade between epidural levobupivacaine and morphine for postoperative analgesia following laparotomy.

Materials and methods

This is a prospective, double-blind, randomized controlled study registered in the Clinical Trials Registry of India (CTRI/2021/04/033102). Ninety patients undergoing elective major abdominal surgery were randomly divided into two groups: levobupivacaine (0.125%/mL) and morphine (0.032 mg/mL) group. All patients received epidural infusion at 6 mL/hour. The visual analog scale (VAS) score at rest and during cough was observed for 24 hours. Heart rate and blood pressure were monitored continuously for 24 hours postoperatively. Additional analgesic requirements, postoperative sedation score, and motor blockade were also compared between the two groups. Statistical analysis was done using the chi-square test, unpaired T-test, and Mann-Whitney test. The sample size estimation was based on a pilot study.

Results

The demographic data and duration of the procedure were comparable in both groups. The initial median VAS score at rest in the levobupivacaine group was high (interquartile range (IQR): 2-4) when compared to the morphine group (IQR: 1-3) at the fourth, sixth, and eighth hour with a P value of <0.05. The initial median VAS score at coughing in the levobupivacaine group was 4 (IQR: 3-5) and in the morphine group was 3 (IQR: 3-4). The VAS score at rest and at coughing was significantly higher in the levobupivacaine group. Heart rate was stable in both groups, and a significant fall in mean arterial blood pressure was observed in the levobupivacaine group. The sedation score was significantly higher in the morphine group (IQR: 2-2) when compared to the levobupivacaine group (IQR: 1-2) at the fourth hour postoperatively with a P value of <0.05. Motor blockade was found to be stronger in the levobupivacaine group (IQR: 0-2) when compared to the morphine group (IQR: 0-0) at the fourth, sixth, and eighth hour postoperatively with a P value of <0.05. An additional dose of fentanyl was required by 6.7% of the patients in the levobupivacaine group and 8.9% of the patients in the morphine group. In the levobupivacaine group, 11.1% reported headaches, 2.2% reported vomiting, and 4.4% reported hypotension, and no pruritus was reported. In the morphine group, 11.1% reported tachycardia, 6.7% reported nausea and vomiting, 6.4% reported pruritus, and 2.2% reported hypotension.

Conclusion

We conclude that patients receiving epidural morphine had better pain scores with better hemodynamic stability than the epidural levobupivacaine group following laparotomy. The morphine group had less motor blockade. Sedation was observed in the morphine group. Additional analgesics were required in both groups. The adverse effects observed in the epidural morphine group were tachycardia, nausea, pruritus, and itching. The epidural levobupivacaine group reported headache, vomiting and fever, and hypotension.

## Introduction

Ineffective pain management following major abdominal surgeries increases the cardiovascular workload by stimulating the neuroendocrine and sympathetic nervous systems, delaying mobilization, and triggering thromboembolic events [1]. The administration of local anesthetics in epidural analgesia improves patient outcomes, with early mobilization leading to early postoperative recovery [2,3].

Epidural anesthesia is frequently utilized to provide analgesia in the intraoperative as well as the postoperative periods for laparotomy [4,5]. Local anesthetics such as bupivacaine, levobupivacaine, and ropivacaine are the preferred medications for epidural anesthesia. However, they may have adverse effects such as hypotension, urinary incontinence, and motor blockade. For epidural anesthesia, bupivacaine is a common local anesthetic medication. Since the S-enantiomer of bupivacaine, levobupivacaine, is less cardiotoxic, we considered it as an epidural local anesthetic in our study. Epidural morphine may be utilized for analgesia, as a supplement to general anesthesia [6]. Because it does not result in hypotension or motor blockade, the opioid medication morphine can be explored for postoperative analgesia via the epidural route [7].

There are no studies published comparing the above two drugs to the best of our knowledge. Hence, we decided to compare epidural levobupivacaine and epidural morphine for postoperative analgesia following major abdominal surgeries. The primary objective of the study was to compare visual analog scale (VAS) scores at rest and at cough between epidural levobupivacaine and epidural morphine for postoperative analgesia following major abdominal surgery. The secondary objectives were to compare the hemodynamic parameters, sedation score, and motor blockade between the two groups.

## Materials and methods

This study was conducted following the Declaration of Helsinki and reported according to the Consolidated Standards of Reporting Trials (CONSORT) statement. Institutional Ethical Committee approval (IEC number 2401) was obtained, and the trial was registered in the Clinical Trials Registry of India (CTRI/2021/04/033102). All patients aged between 18 and 70 years, belonging to the American Society of Anesthesiologists (ASA) grade I, II, and III, with body mass index (BMI) ranging between 18.5 and 24.9 kg/m^2^ undergoing elective major abdominal surgeries were included in the study. The exclusion criteria included pregnancy, patients with drug allergies, patients on preoperative opioids, patient refusal, uncooperative patients, systemic or local infection, and patients with cardiac disease, severe lung disease, liver disease, and kidney disease.

We obtained informed consent from all patients. Eligible patients were recruited consecutively and were randomized using a computer-generated random sequence in varying block sizes of four. The random allocation sequence was generated by the support staff of the institute who was not part of the study. The location sequence was used to make sequentially numbered opaque sealed envelopes. On inclusion of a patient, the principal investigator would open one envelope and allocate the patient to either of the arms, either to receive levobupivacaine (group L) or morphine (group M) epidurally. He would prepare the drug solution according to the group involved and took no further part in the study. The patients and the anesthesiologist who performed epidural catheterization and collected intraoperative and postoperative information were also blinded to the treatment allocation.

An 18-Gauge intravenous (IV) cannula was inserted the previous night, and infusion with 0.9% normal saline was started at a rate of 100 mL/hour. A pulse oximeter, noninvasive blood pressure cuff, and electrocardiogram were connected, and additional intravenous (IV) access was established. With the patient in the sitting position, epidural catheterization was performed in all patients before surgery, at the T12-L1 intervertebral space. Once the space was identified and the catheter was fixed with 4 cm of catheter inside the epidural space, a test dose of 3 mL 1.5% lignocaine with 1:200,000 adrenaline drug was given. A 6 mL bolus of 0.25% levobupivacaine in the levobupivacaine group (group L) and a 6 mL bolus of 3 mg morphine in the morphine group (group M) were given before the skin incision. After which, continuous epidural infusion at 6 mL/hour was started in each group with dilution as follows: group L, 25 mL of 0.25% levobupivacaine diluted to 50 mL to make it 0.125% levobupivacaine, and group M, 1 mL of morphine containing 10 mg diluted to 10 mL, and 1.8 mL of this dilution was further diluted to 50 mL, with each milliliter containing 0.032 mg of morphine (0.192 mg/hour). A pilot study was conducted on 10 patients using the VAS score at rest as a parameter. The VAS score in the levobupivacaine group was 3.2±0.6, and in the morphine group, it was 2.5±0.8. For the study to have 80% power and alpha error at 0.05, the sample size for our study was derived as N=42 in each group. Assuming 10% dropouts, the total was taken as 45 in each group.

The patients were given general anesthesia with fentanyl 2 mcg/kg as intraoperative analgesia, thiopentone 2.5% as induction agent, and 0.1 mg/kg vecuronium as muscle relaxant. Sevoflurane (1%-2%) and a combined 50% O2 + 50% N2O were used to maintain the depth of anesthesia. All patients received urinary catheters. The mean blood pressure and heart rate were recorded at baseline and intraoperatively. The patients were monitored continuously and recorded every 30 minutes from baseline until four hours and every two hours up to 24 hours. After the conclusion of surgery, patients were extubated after the administration of reversal agents and shifted to the postanesthetic care unit (PACU). The patients were connected to an infusion pump according to their groups, and epidural infusion was started at the rate of 6 mL/hour for 24 hours depending on hemodynamic parameters.

The intensity of postoperative pain at rest and at coughing was assessed using the visual analog scale (VAS) with 0 being no pain and 10 hurting worst [8]. An epidural bolus of 4 mL was given as rescue analgesia if the VAS score was ≥3. If the pain was persistent, an injection of fentanyl 0.5 mcg/kg was given intravenously. The total doses of analgesic consumed were recorded. The motor blockade was evaluated using the Bromage scale: 0, no motor block; 1, inability to flex knees 30°; 2, inability to flex knees and ankle; and 3, complete motor block. The sedation score was evaluated using the Ramsay sedation score: 1, the patient is anxious, agitated, restless, or both; 2, the patient is cooperative, oriented, and tranquil; 3, the patient responds to verbal commands; 4, brisk response to a light glabellar tap or loud auditory stimulus; 5, sluggish response to a light glabellar tap or loud auditory stimulus; and 6, no response to a light glabellar tap or loud auditory stimulus.

The incidence of adverse effects such as nausea, vomiting, respiratory depression, pruritus, bradycardia, and hypotension was recorded in the postoperative period. Hypotension was interpreted as a fall of the initial value of systolic blood pressure by more than 20%, and bradycardia is interpreted as a heart rate of <20% of the baseline. If there was a fall in blood pressure, the crystalloid infusion was increased; if hypotension persisted, patients were given ephedrine 6 mg intravenously. Vomiting, nausea, and pruritus were treated with an ondansetron intravenous injection. The patients were monitored in the PACU for 24 hours. The severity of complications such as nausea and vomiting was noted and assessed using scores for postoperative nausea (0, no nausea; 1, mild nausea (not requiring rescue antiemetic); 2, moderate nausea (requires rescue antiemetic); and 3, severe nausea (resistant to treatment)) and vomiting (0, no vomiting; 1, mild vomiting (not requiring rescue antiemetic); 2, vomiting (requires rescue antiemetic); and 3, vomiting (resistant to treatment)). The rescue antiemetic administered was ondansetron injection 4 mg intravenously.

Data were entered in Microsoft Excel (Microsoft Corp., Redmond, WA, USA) and analyzed using Statistical Package for the Social Sciences (SPSS) version 20.0 (IBM SPSS Statistics, Armonk, NY, USA). Descriptive statistics such as mean, standard deviation, median, and interquartile range (IQR) for quantitative variables were used to describe the data. Inferential statistics such as the chi-square test were used to differentiate the proportions. The unpaired T-test and Mann-Whitney test were used to find the difference in the means between the levobupivacaine and morphine groups. A P value of less than 0.05 was considered significant.

## Results

**Figure 1:**
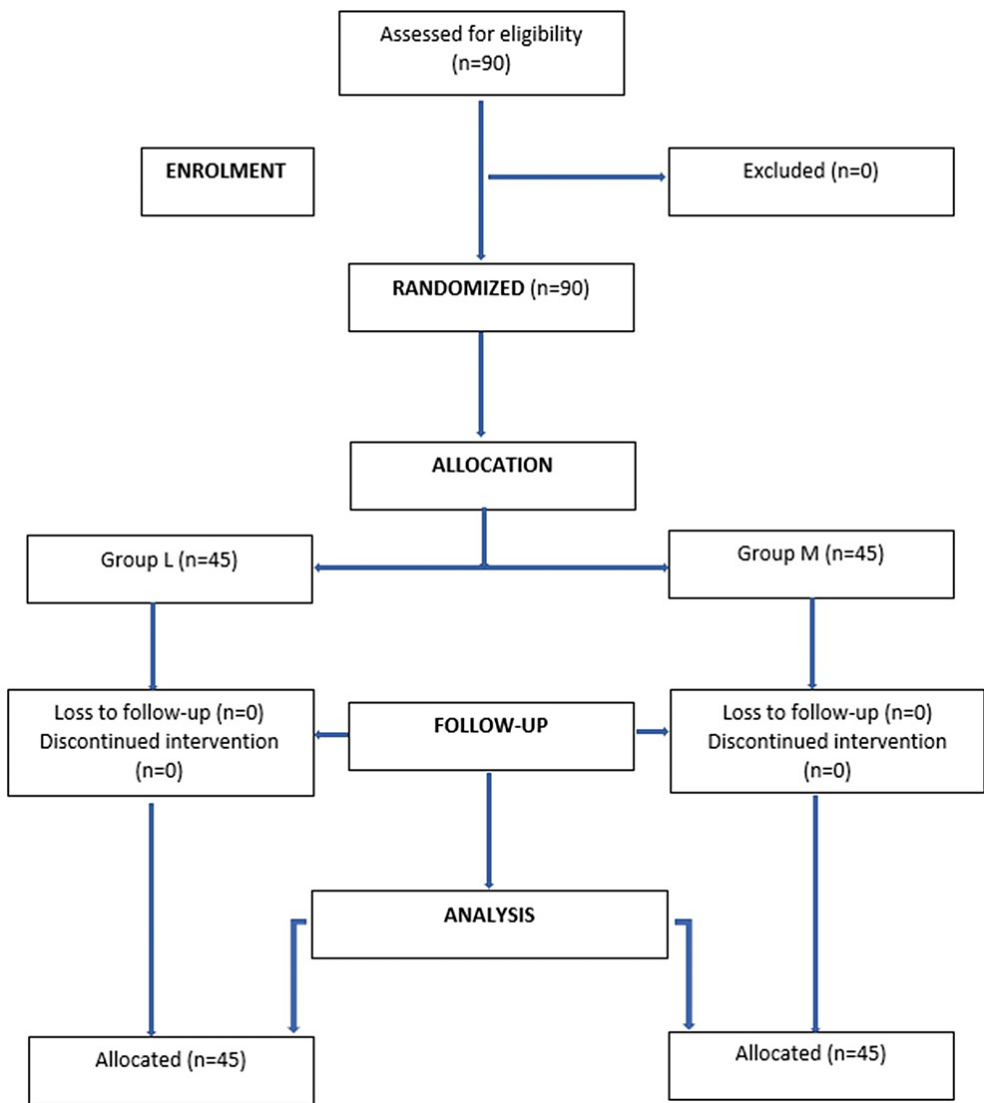
CONSORT flowchart CONSORT: Consolidated Standards of Reporting Trials

The Consolidated Standards of Reporting Trials flow diagram showing the flow of participants in the study is depicted in Figure 1.

There were no significant changes concerning the demographic profile and duration of surgery in the groups (Table 1).

**Table 1:** Basic characteristics of the study participants *P value is not statistically significant (>0.05). Values are in mean±standard deviation, and P values were obtained using unpaired t-test and chi-square test. M/F: male/female, ASA: American Society of Anesthesiologists

Parameters	Group L	Group M	T-test	P value
Age (years)	47.82±11.43	45.09±9. 38	1.10	0.218*
Gender (M/F)	17/28	23/22	1.62	0.203*
ASA (II/III)	9/36	8/37	0.07	0.788*
Body mass index (kg/m^2^)	21.52±1. 96	22.17±1. 63	2.77	0.091*
Duration of open surgery (minutes)	191.11±27.15	188. 44±27.76	0.92	0.253*

The VAS score at rest was statistically significant at four, six, eight, 12, and 20 hours with a median (interquartile range) of 3 (2-4), 2 (1.5-3), 2 (0.5-2.5), 0 (0-2), and 0 (0-0) in group L and 2 (1-3), 2 (0-3), 1 (0-2), 0 (0-0), and 0 (0-0) in group M, respectively, with P values of 0.027, 0.012, 0.027, 0.016, and 0.042, respectively, which was statistically significant. All other times had insignificant P values concerning the VAS score at rest (Table 2).

**Table 2:** VAS scores at rest at various timelines among the study participants P values are obtained using the Mann-Whitney U test. †P value is statistically significant (<0. 05). *P value is not statistically significant (>0.05). VAS: visual analog scale, IQR: interquartile range

VAS score at rest (hours)	Group L		Group M		Z score	P value
	Median VAS score (n=45)	IQR	Median VAS score (n=45)	IQR		
2	3.0	3-4	3.0	2-4	-1.49	0.137*
4	3.0	2-4	2.0	1-3	-2.21	0.027†
6	2.0	1.5-3	2.0	0-3	-2.50	0.012†
8	2.0	0.5-2.5	1.0	0-2	-2.21	0.027†
10	1.0	0-2	0.0	0-1	-1.87	0.062*
12	0.0	0-2	0.0	0-0	-2.42	0.016†
14	0.0	0-1	0.0	0-0	-1.66	0.097*
16	0.0	0-1	0.0	0-0.5	-0.62	0.535*
18	0.0	0-0	0.0	0-0	-0.02	0.836*
20	0.0	0-0	0.0	0-0	-2.03	0.042†
22	0.0	0-0	0.0	0-0	0.00	1.000*
24	0.0	0-0	0.0	0-0	0.00	1.000*

The VAS score at coughing was statistically significant at eight, 14, 16, 20, 22, and 24 hours with a median (interquartile range) of 2 (0-4), 0 (0-2), 0 (0-1), 0 (0-0), 0 (0-1), and 0 (0-0) in group L and 1 (0-2), 0 (0-0), 0 (0-0), 0 (0-0), 0 (0-0), and 0 (0-0), in group M, respectively, with P values of 0.046, 0.011, 0.009, 0.003, 0.042, and 0.042, respectively, which was statistically significant. All other times had insignificant P values concerning the VAS score at coughing (Table 3).

**Table 3:** VAS scores at coughing at various timelines among the study participants P values are obtained using the Mann-Whitney U test. †P value is statistically significant (<0.05). *P value is not statistically significant (>0.05). VAS: visual analog scale, IQR: interquartile range

VAS score at coughing (hours)	Group L		Group M		Z score	P value
	Median VAS score (n=45)	IQR	Median VAS score (n=45)	IQR		
2	4.0	3-5	3.0	3-4	-1.49	0.136*
4	4.0	2.5-4	3.0	2-4	-1.55	0.122*
6	3.0	1.5-4	2.0	0-3	-1.85	0.065*
8	2.0	0-4	1.0	0-2	-1.99	0.046†
10	2.0	0-3	1.0	0-2	-1.63	0.103*
12	1.0	1-2	0.0	0-1	-1.52	0.123*
14	0.0	0-2	0.0	0-0	-2.54	0.011†
16	0.0	0-1	0.0	0-0	-2.61	0.009†
18	0.0	0-0.5	0.0	0-0	-1.08	0.281*
20	0.0	0-0	0.0	0-0	-2.94	0.003†
22	0.0	0-0	0.0	0-0	-2.03	0.042†
24	0.0	0-0	0.0	0-0	-2.03	0.042†

**Figure 2:**
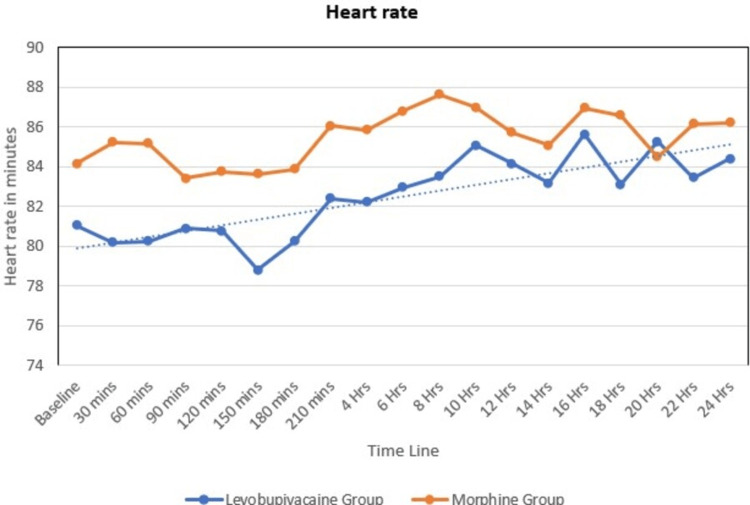
Line diagram showing heart rate distribution at various timelines among the study participants mins: minutes, Hrs: hours

The heart rates of the study participants in both groups showed an inclining trend. The heart rate was more in group M than in group L at 60 minutes and was statistically significant (Figure 2).

**Figure 3:**
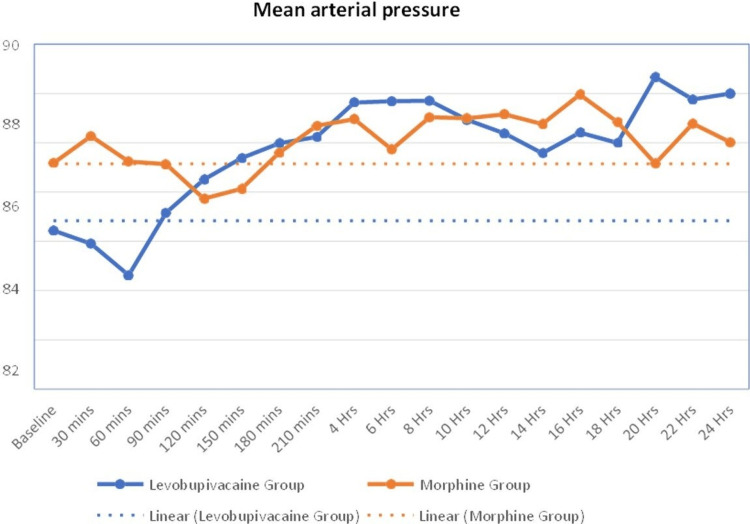
Line diagram showing mean arterial pressure distribution at various timelines among the study participants mins: minutes, Hrs: hours

The mean arterial pressure of the study participants in the levobupivacaine group shows an inclining trend, and in the morphine group, it shows a constant direction. The mean arterial pressure was similar over various periods except at baseline and at 60 minutes (group L: 80.62±10.67, group M: 85.24±11.16). The P value was found to be 0.048 in both groups (Figure 3).

Motor blockade was statistically significant over four, six, and eight hours between the groups (Table 4).

**Table 4:** Motor blockade distribution at various timelines among the study participants P values are obtained using the Mann-Whitney U test. †P value is statistically significant (<0.05). *P value is not statistically significant (>0.05). VAS: visual analog scale, IQR: interquartile range, NA: not assessed

Motor blockade	Levobupivacaine group			Morphine group			Z value	P value
	Median motor blockade score (n=45)	IQR	Minimum-maximum value	Median VAS score (n=45)	IQR	Minimum-maximum value		
2 hours	NA	NA	NA	NA	NA	NA	NA	NA
4 hours	0.0	0-0	0-2	0.0	0-0	0-0	-3.33	0.001†
6 hours	0.0	0-0	0-2	0.0	0-0	0-0	-2.94	0.003†
8 hours	0.0	0-0	0-2	0.0	0-0	0-0	-2.03	0.042†
10 hours	0.0	0-0	0-1	0.0	0-0	0-0	-1.75	0.080*
12 hours	0.0	0-0	0-1	0.0	0-0	0-0	-1.42	0.155*
14 hours	0.0	0-0	0-1	0.0	0-0	0-0	-1.00	0.317*
16 hours	0.0	0-0	0-0	0.0	0-0	0-0	0.0	1.000*
18 hours	0.0	0-0	0-0	0.0	0-0	0-0	0.0	1.000*
20 hours	0.0	0-0	0-0	0.0	0-0	0-0	0.0	1.000*
22 hours	0.0	0-0	0-0	0.0	0-0	0-0	0.0	1.000*
24 hours	0.0	0-0	0-0	0.0	0-0	0-0	0.0	1.000*

Motor blockade was found to be more in group L when compared to group M. The sedation score was statistically significant at four hours with a median (interquartile range) of 0 (2-2) both in group L and group M with a P value of 0.014, which was statistically significant. The sedation score was more in the morphine group when compared to the levobupivacaine group (Table 5).

**Table 5:** Sedation score distribution at various timelines among the study participants P values are obtained using the Mann-Whitney U test. †P value is statistically significant (<0.05). *P value is not statistically significant (>0.05). IQR: interquartile range

Sedation score	Levobupivacaine group			Morphine group			Z value	P value
	Median sedation score (n=45)	IQR (25^th^-75^th ^percentile)	Minimum-maximum value	Median sedation score (n=45)	IQR (25^th^-75^th ^percentile)	Minimum-maximum value		
2 hours	2.0	0 (2-2)	2-2	2.0	0 (2-2)	2-2	0.00	1.000*
4 hours	2.0	0 (2-2)	2-3	2.0	0 (2-2)	2-3	-2.45	0.014†
6 hours	2.0	0 (2-2)	2-3	2.0	0 (2-2)	2-3	-1.49	0.136*
8 hours	2.0	0 (2-2)	2-3	2.0	0 (2-2)	2-3	-1.49	0.136*
10 hours	2.0	0 (2-2)	1-3	2.0	0 (2-2)	1-3	-0.01	0.985*
12 hours	2.0	0 (2-2)	2-2	2.0	0 (2-2)	2-2	0.00	1.000*
14 hours	2.0	0 (2-2)	2-2	2.0	0 (2-2)	2-3	-1.00	0.317*
16 hours	2.0	0 (2-2)	2-2	2.0	0 (2-2)	2-2	0.00	1.000*
18 hours	2.0	0 (2-2)	2-2	2.0	0 (2-2)	2-2	0.00	1.000*
20 hours	2.0	0 (2-2)	2-2	2.0	0 (2-2)	2-2	0.00	1.000*
22 hours	2.0	0 (2-2)	2-2	2.0	0 (2-2)	2-2	0.00	1.000*
24 hours	2.0	0 (2-2)	2-2	2.0	0 (2-2)	2-2	0.00	1.000*

The additional fentanyl doses required among the levobupivacaine group was 6.7% (three patients), and the morphine group was 8.9% (four patients). The distribution of the additional fentanyl dose required was found to be similar in both groups with a P value of more than 0.05. The following adverse effects were observed in the levobupivacaine group: hypotension (4.4%) and vomiting (2.2%). Similarly, in the morphine group, the following were observed: nausea (6.7%) and pruritus/itching (6.4%). Urinary retention could not be assessed as all patients were catheterized.

## Discussion

Local anesthetics that are used for epidural analgesia cause significant changes in hemodynamic parameters [9]. Our trial demonstrates the superiority of epidural morphine provided by the better VAS scores at rest and at coughing, with better hemodynamic parameters compared to that of the epidural levobupivacaine in major abdominal surgery. Motor blockade was seen over two and four hours in the epidural levobupivacaine group. The morphine group required higher additional fentanyl doses when compared with the other group. Adverse effects such as hypotension were reported by the levobupivacaine group, whereas nausea, pruritus, and itching were reported by the morphine group.

In this study, individuals’ median VAS scores at rest and at coughing both showed a declining trend in both groups. Similar results were shown by Türkoğlu et al. [10], where VAS scores were considerably higher in the levobupivacaine group compared with the levobupivacaine plus morphine and the levobupivacaine plus tramadol groups. In contrast, Casati et al. [11] assessed the effectiveness of pain management and the level of motor blockage with an epidural infusion of 0.125% levobupivacaine and IV patient-controlled analgesia (PCA) morphine and demonstrated lower VAS values in the levobupivacaine group when compared with the IV PCA morphine group.

Levobupivacaine concentrations of 1.5 mg/dL and 5 mg/dL were compared in research by Dernedde et al. [12] in postoperative patient-controlled epidural analgesia. During the first 24 hours, arterial blood pressure was somewhat lower in the 5 mg/mL group, which was similarly shown in our trial in the levobupivacaine group. The cause is the propensity for hypotension that local anesthetics have. In line with our observations, the results of the study by Egashira et al. [13], who examined the efficacy of epidural ropivacaine versus epidural levobupivacaine, indicated that neither the epidural 0.125% levobupivacaine group nor the 0.2% ropivacaine group had significantly different arterial blood pressure or heart rates. While a study by Singelyn et al. [14] concluded that epidural analgesia with morphine offers greater pain relief and expedites knee rehabilitation.

Levobupivacaine was studied by De Cosmo et al. [15] to determine its efficacy in treating post-thoracotomy pain. Patients in this trial who received 0.125% levobupivacaine had a low incidence of nausea, vomiting, and pruritus. Epidural levobupivacaine at a concentration of 0.125% in combination with sufentanil provided effective pain management with no side effects and no motor block at all, which was similarly explained in this study.

This study has a few limitations. First, we did not measure the sensory blockade in both groups. Second, the postoperative mortality and length of hospital stay were not recorded. However, all patients were followed up until the time of discharge. Since we were comparing local anesthetic with opioid epidurally, the addition of fentanyl to levobupivacaine would have acted as a confounding factor in our study. Hence, we did not use the above combination of drugs.

## Conclusions

The patients receiving epidural morphine had better pain scores with better hemodynamic stability than the epidural levobupivacaine group following laparotomy. The morphine group had less motor blockade. Sedation was observed in the morphine group. Additional analgesics were required in both groups. The adverse effects observed in the epidural morphine group were tachycardia, nausea, and pruritus. The epidural levobupivacaine group reported headache, vomiting and fever, and hypotension.

We conclude that epidural morphine provides better pain relief with less motor blockade than epidural levobupivacaine following major abdominal surgeries. The levobupivacaine group had hypotension and motor blockade, and the morphine group had nausea, vomiting, and pruritus, but their side effects did not affect the patient’s comfort and were treatable.

## References

[1] Whiteside JB, Wildsmith JA (2001). Developments in local anaesthetic drugs. Br J Anaesth.

[2] Garimella V, Cellini C (2013). Postoperative pain control. Clin Colon Rectal Surg.

[3] Moraca RJ, Sheldon DG, Thirlby RC (2003). The role of epidural anesthesia and analgesia in surgical practice. Ann Surg.

[4] Avila Hernandez AN, Singh P (2022). Epidural anesthesia. https://www.ncbi.nlm.nih.gov/books/NBK542219/.

[5] (2023). NYSORA: Epidural anesthesia and analgesia. https://www.nysora.com/topics/regional-anesthesia-for-specific-surgical-procedures/abdomen/epidural-anesthesia-analgesia/.

[6] Gottschalk A, Poepping DM (2015). [Epidural analgesia in combination with general anesthesia]. Anasthesiol Intensivmed Notfallmed Schmerzther.

[7] Hurley RW, Elkassabany NM, Wu CL (2010). Acute postoperative pain. Miller's anesthesia, 7th edition.

[8] Venkatraman R, Abhinaya RJ, Sakthivel A, Sivarajan G (2016). Efficacy of ultrasound-guided transversus abdominis plane block for postoperative analgesia in patients undergoing inguinal hernia repair. Local Reg Anesth.

[9] Reschke MM, Monks DT, Varaday SS, Ginosar Y, Palanisamy A, Singh PM (2020). Choice of local anaesthetic for epidural caesarean section: a Bayesian network meta-analysis. Anaesthesia.

[10] Türkoğlu Z, Karacaer F, Biricik E, Ilgınel M, Ünlügenç H (2019). Comparison of the effects of epidural levobupivacaine with tramadol or morphine addition on postoperative analgesia following major abdominal surgery. Turk J Anaesthesiol Reanim.

[11] Casati A, Ostroff R, Casimiro C (2008). 72-hour epidural infusion of 0.125% levobupivacaine following total knee replacement: a prospective, randomized, controlled, multicenter evaluation. Acta Biomed.

[12] Dernedde M, Stadler M, Bardiau F, Boogaerts JG (2005). Comparison of 2 concentrations of levobupivacaine in postoperative patient-controlled epidural analgesia. J Clin Anesth.

[13] Egashira T, Fukasaki M, Araki H, Sakai A, Okada M, Terao Y, Hara T (2014). Comparative efficacy of levobupivacaine and ropivacaine for epidural block in outpatients with degenerative spinal disease. Pain Physician.

[14] Singelyn FJ, Deyaert M, Joris D, Pendeville E, Gouverneur JM (1998). Effects of intravenous patient-controlled analgesia with morphine, continuous epidural analgesia, and continuous three-in-one block on postoperative pain and knee rehabilitation after unilateral total knee arthroplasty. Anesth Analg.

[15] De Cosmo G, Mascia A, Clemente A, Congedo E, Aceto P (2005). Use of levobupivacaine for the treatment of postoperative pain after thoracotomies. Minerva Anestesiol.

